# Parent-child skin carotenoid level and vegetable intake relationships in users of children’s cafeterias in Japan

**DOI:** 10.3389/fnut.2024.1388233

**Published:** 2024-08-14

**Authors:** Kayo Kurotani, Kazunori Ohkawara, Hidemi Takimoto

**Affiliations:** ^1^Graduate School of Life Sciences, Showa Women’s University, Setagaya-ku, Japan; ^2^Graduate School of Informatics and Engineering, University of Electro-Communications, Chofu, Japan; ^3^National Institute of Biomedical Innovation, Health and Nutrition, Settsu, Japan

**Keywords:** skin carotenoid, pressure-mediated reflection spectroscopy, familial resemblance, children’s cafeteria, vegetable intake, Japanese

## Abstract

**Introduction:**

Studies on the relationship between parental and child dietary intakes are limited in Asian populations. Here, we examined parent-child relationships in skin carotenoid levels and vegetable intake in a Japanese community.

**Methods:**

The study participants were 58 children aged 6–15 years and 39 of their guardians (parents) using children’s cafeterias. Skin carotenoid levels were measured using the Veggie Meter^®^, and the number of vegetable dishes (equivalent to a serving of 70 g) was evaluated using a self-administered questionnaire.

**Results:**

The mean (standard deviation; SD) skin carotenoid levels were 366.8 (74.0) in children and 315.0 (101.4) in parents. The partial correlation coefficient between parents’ and children’s skin carotenoid levels, adjusting for cafeteria, sex, parental dietary supplement use, and household financial status, was 0.38 (*P* = 0.02); after adjustment for smoking status and BMI, the positive correlation was attenuated (*r* = 0.25, *P* = 0.14). A positive correlation was observed between parents’ and children’s vegetable dish intake (*r* = 0.30, *P* = 0.02).

**Conclusion:**

This cross-sectional study identified a positive correlation between parent-child intake of vegetable dishes, accounting for potential confounders. However, the positive correlation observed between parent-child skin carotenoid levels may have been attenuated by internal factors such as smoking and obesity.

## 1 Introduction

Early childhood is a crucial period for the development of dietary behaviors. These behaviors established during childhood are likely to persist into adulthood (1); exposure to vegetables at an early age has been associated with higher consumption of vegetables in adulthood (1). Additionally, recent systematic (2) and narrative reviews (3) have shown that parental dietary behaviors influence children’s eating habits. A pooled meta-analysis of 45 studies, mainly from Western countries, reported weak to moderate parent-child dietary intake associations (2), with a pooled correlation coefficient for fruit and vegetable intake in parents and children of 0.29 (95% CI: 0.26–0.33) (2). However, studies on the association between parental and child dietary intake in Asian populations are limited. There is a possibility of differences in the parent-child resemblance of dietary behaviors between Asian and Western populations (4).

Various spectroscopy technologies have been employed to identify and quantify carotenoids in the skin, including resonance Raman spectroscopy (RRS) and pressure-mediated reflection spectroscopy (RS). RRS has demonstrated increased accuracy and precision in determining skin carotenoids compared with RS (5). Meanwhile, RS has produced moderate to strong correlations with serum/plasma carotenoid status (5, 6), a critical biomarker of vegetable and fruit intake (7). RS can be applied across different ages, sexes, and ethnicities (8–11). This noninvasive optical method enables the rapid quantitative assessment of carotenoids, showing potential for applications in nutritional epidemiology (5). Skin carotenoid measurement offers a more convenient and noninvasive approach for estimating fruit and vegetable consumption compared to traditional methods, such as handwritten 3-day dietary records (12). There is currently limited information on skin carotenoid level relationships between parents and children.

Here, we examined the association between parent-child skin carotenoid levels and vegetable intake in a Japanese community. Identifying the parent-child resemblances in skin carotenoid levels and vegetable intake within a community population may provide evidence supporting initiatives in food education, leading to enhanced consumption of vegetables and fruits among both parents and children.

## 2 Materials and methods

### 2.1 Study population

This study included children aged 6–15 years and their guardians (parents) who attended two children’s cafeterias in Tokyo, Japan, between November and December 2019. A children’s cafeteria is a free or low-cost cafeteria where a child can visit alone (13). Children’s cafeterias support children suffering from poverty by providing free or low-cost meals in a comfortable environment and are bases for multi-generational community communication where local children and adults eat together (13). Children’s cafeterias were held twice a month, and one cafeteria supplied meals to approximately 100 children and their parents; another supplied meals to approximately 30 children and their parents.

Written informed consent was obtained from all the parents. Measurements of skin carotenoid levels were conducted among children who agreed to participate in the study. A self-administered questionnaire was conducted separately with children and their parents. Parents who did not visit the children’s cafeteria during the study period completed the questionnaire at home. Trained staff supported small children in completing the questionnaires. Sixty children and 51 parents participated in this study; of these, we excluded two children and 12 parents for whom skin carotenoid levels were not measured, resulting in a final analysis of 58 children and 39 parents. This study was conducted in accordance with the Declaration of Helsinki and the Ethical Guidelines for Medical and Health Research Involving Human Subjects. The study protocol was approved by the Ethics Committee of the National Institute of Biomedical Innovation, Health, and Nutrition (KENEI 118).

### 2.2 Measurement of skin carotenoid levels

Skin carotenoid levels were measured using the Veggie Meter^®^ (Longevity Link Corporation) (14). The Veggie Meter^®^ is a validated, research-grade instrument that utilizes RS to estimate carotenoid concentration in the skin. Calibration with the supplied dark and white reference materials was performed hourly. The participants wiped their fingers using disposable towels before measurement, inserted the middle finger of their left hand into the device’s finger cradle, and their fingertip was pressed against the convex contact lens surface with the assistance of a spring-loaded lid. Triplicate measurements were performed to minimize inter-individual and intra-individual variability (15). The skin carotenoid score ranged from 0 to 1200 and was unitless.

### 2.3 Dietary intake

The number of vegetable dishes consumed per day was assessed using a self-administered questionnaire. Vegetable dishes were defined as 70 g of vegetables based on the Japanese Food Guide Spinning Top (16) and were assessed across four categories: almost none, 1–2 dishes/day, 3–4 dishes/day, and ≥ 5 dishes/day among parents; and six categories: almost none, 1 dish/day, 2 dishes/day, 3 dishes/day, 4 dishes/day, and ≥ 5 dishes/day among children (17, 18). Examples of 70 grams of vegetables were shown in the questionnaire. Dietary supplement use was assessed for only parents across four categories: every day, sometimes, rarely, and none. However, we did not assess the type of dietary supplement used. The number of well-balanced meals, including staple, main, and vegetable dishes, consumed per day was also assessed.

### 2.4 Demographics and lifestyle factors

Demographic and lifestyle factors were assessed using self-administered questionnaires. Information on sex and age was collected from both children and parents. Information on self-reported height and weight, smoking status (non-smoker or current smoker), alcohol drinking status (rarely, 1-3 days/month, or ≥ 1 day/week), final educational level (high school, college, university, or graduate school), current job (full-time employment, part-time job, self-employed, or unemployed), regular exercise (almost every day, 2-5 days/week, or 1 day/week), and household financial status (comfortable, neutral, or uncomfortable) was gathered from parents. Body mass index (BMI) was calculated by dividing weight (kg) by the square of height (m) (kg/m^2^).

### 2.5 Statistical analysis

Pearson’s partial correlation coefficient was used to assess the association between parental skin carotenoid levels and their children. Model 1 was adjusted for the specific children’s cafeteria visited (A and B), sex, parental dietary supplement use, and household financial status, while Model 2 also incorporated adjustments for parental smoking status. Moreover, parental BMI was included as an additional adjustment in Model 3. We also examined the correlation between parental and child vegetable dish intake using Spearman’s partial correlation coefficient. Vegetable dish intake categories were combined into four groups for both children and parents: almost none, 1–2 dishes/day, 3–4 dishes/day, and ≥ 5 dishes/day. Sensitivity analysis was performed for the mother because the mother-child correlation may be different from the parent-child correlation. We could not conduct an analysis for fathers, as the sample of fathers was small (*n* = 4). Furthermore, the association between skin carotenoid level and vegetable dish intake among both children and parents was assessed using a regression model. For the regression analysis, the vegetable dish categories were adjusted based on the distribution of the number of vegetable dishes consumed by children and parents: almost none, 1 dish/day, 2 dishes/day, and ≥ 3 dishes/day among children: and almost none or 1–2 dishes/day and ≥ 3 dishes/day among parents. All statistical analyses were performed using STATA (version 17.0; StataCorp). Statistical significance was set at P < 0.05.

## 3 Results

Participants’ characteristics are listed in Table 1. A high proportion of children were elementary school age and had good subjective health. Parents were more likely to be female, non-smokers, and employed. The mean age and BMI were 44.4 (standard deviation; SD = 5.2) years and 22.1 (3.2) kg/m^2^, respectively. The number of well-balanced meals was higher in children than in parents; the proportion of participants who had well-balanced meals twice per day or more was 77.6% in children and 41.0% in parents. However, the proportion of participants who had almost no vegetable dishes per day was higher in parents than in children (12.1 vs. 2.6%). Fifteen percent of parents used dietary supplements every day. The mean (SD) skin carotenoid levels were 366.8 (74.0) in children and 315.0 (101.4) in parents.

**TABLE 1:** Characteristics of study participants.

	Children (*n* = 58)		Parents (*n* = 39)	
	***n***	**(%)**	***n***	**(%)**
Women [*n* (%)]	31	(53.4)	35	(89.7)
**School grade [n (%)]**				
Elementary school grades 1-2	21	(36.2)		
Elementary school grades 3-4	27	(46.6)		
Elementary school grades 5-6	8	(13.7)		
Junior high school	2	(3.4)		
Age (year; mean (SD))	–	–	41.4	(5.2)
Body mass index (kg/m^2^; mean (SD))^1^	–	–	22.1	(3.2)
**Household financial status (n (%)) ^1^**				
Comfortable	–	–	14	(36.8)
Neutral	–	–	17	(44.7)
Uncomfortable	–	–	7	(18.4)
**Parental educational level (n (%))**				
High school	–	–	10	(25.6)
College	–	–	11	(28.2)
University	–	–	18	(46.2)
Graduate school	–	–	0	(0.0)
**Parental employment (n (%))**				
Regular employment	–	–	16	(41.0)
Part-time job	–	–	12	(30.8)
Self-employed	–	–	4	(10.3)
No job	–	–	7	(17.9)
**Parental smoking status (n (%))^1^**				
Non-smoker	–	–	32	(84.2)
Current smoker	–	–	6	(15.8)
**Parental alcohol drinking status [n (%)]^1^**				
Rarely	–	–	15	(39.5)
1-3 days/month	–	–	8	(21.1)
≥ 1 day/week	–	–	15	(39.5)
**Parental regular exercise [n (%)]^1^**				
Almost everyday	–	–	3	(7.7)
2-5 days/week	–	–	5	(12.8)
1 day/week	–	–	11	(28.2)
Rarely	–	–	20	(51.3)
**Subjective health status [n (%)]**				
Very good/Good	52	(89.7)	27	(71.1)
Neutral	4	(6.9)	5	(13.2)
Bad/Very bad	2	(3.4)	6	(15.8)
**Parental dietary supplement use [n (%)]^1^**				
Every day	–	–	6	(15.8)
Sometimes	–	–	8	(21.0)
Rarely	–	–	3	(7.9)
None	–	–	21	(55.3)
**Number of well-balanced meals/day [n (%)]^2^**				
0	7	(12.1)	5	(12.8)
1	6	(10.3)	18	(46.2)
2	28	(48.3)	10	(25.6)
3	17	(29.3)	6	(15.4)
**Number of vegetable dishes/day (%)^1^,^3^**				
Almost none	7	(12.1)	1	(2.6)
1-2 dishes	40	(69.0)	18	(47.4)
3-4 dishes	11	(19.0)	19	(50.0)
≥ 5 dishes	0	(0.0)	1	(2.6)
Skin carotenoid levels [mean (SD)]	366.8	(74.0)	315.0	(101.4)

^1^Number of participants was 32 for body mass index, and 38 for smoking status, alcohol drinking, exercise, dietary supplement use, and vegetable dish intake.

^2^Well-balanced meals include staple, main, and vegetable dishes.

^3^Vegetable dishes were defined as 70 *g* of vegetables.

The associations between parental and child skin carotenoid levels are shown in Table 2. In Model 1, the partial correlation coefficient was 0.38 (*P* = 0.02). However, the correlation was attenuated after adjusting for parental smoking status and BMI in Model 3 (*r* = 0.25, *P* = 0.14). The association between parental and child vegetable dish intake was statistically significant in Model 3 (*r* = 0.30, *P* = 0.02).

**TABLE 2:** Partial correlation coefficients between parents’ and children’s skin carotenoid levels and their vegetable dish intake.

	Skin carotenoid levels		Vegetable dish intake	
	*r*	*P*	*r*	*P*
Model 1^1^	0.38	0.02	0.30	0.02
Model 2 ^2^	0.30	0.07	0.30	0.02
Model 3 ^3^	0.25	0.14	0.30	0.02

^1^Model 1: Adjusted for children’s cafeterias, sex, parental dietary supplement use, and household financial status.

^2^Model 2: Additionally adjusted for parental smoking status.

^3^Model 3: Additionally adjusted for parental BMI.

In the sensitivity analysis, similar associations were observed between the children’s and their mothers’ skin carotenoid levels (Table 3; *r* = 0.26, *P* = 0.14; Model 3). An association between children’s and their mothers’ vegetable dish intake was also observed (*r* = 0.35, *P* = 0.009).

**TABLE 3:** Partial correlation coefficients between mothers’ and children’s skin carotenoid levels and their vegetable dish intake.

	Skin carotenoid levels		Vegetable dish intake	
	*r*	*P*	*r*	*P*
Model 1 ^1^	0.38	0.02	0.35	0.009
Model 2 ^2^	0.31	0.08	0.35	0.009
Model 3 ^3^	0.26	0.14	0.35	0.009

^1^Model 1: Adjusted for children’s cafeterias, children’s sex, mother’s dietary supplement use, and household financial status.

^2^Model 2: Additionally adjusted for smoking status.

^3^Model 3: Additionally adjusted for BMI.

For children, the mean (95% CI) of skin carotenoid levels were 324 (251–397), 370 (245–395), 361 (300–423), and 390 (340–440) for those who had almost no vegetable dishes, 1 dish/day, 2 dishes/day, and ≥ 3 dishes/day, respectively (*P* for trend = 0.15). For parents, the mean (95% CI) of skin carotenoid levels were 287 (249–325) and 326 (282–370) for those who had almost no vegetable dishes or 1–2 dishes/day, and ≥ 3 dishes/day, respectively (*P* for trend = 0.17).

## 4 Discussion

To the best of our knowledge, this is the first study to examine the correlation in skin carotenoid levels between parents and their children. A positive association was found between parental and child skin carotenoid levels as well as vegetable dish intake; however, after considering potential confounding factors, including smoking status and BMI, the positive correlation between parent-child skin carotenoid levels was attenuated. The present parent-child and mother-child positive correlations of vegetable dish intake were similar to those of a recent meta-analysis of 45 studies that examined the association between parent-child resemblance in fruit and vegetable intake (2). The pooled estimated correlation coefficient in this previous meta-analysis was 0.29 [95% confidence interval (CI): 0.26, 0.33]. Sub-group analysis of mother-child pairs from the meta-analysis revealed a similar association (*r* = 0.31; 95% CI: 0.26, 0.36).

We identified a positive association between parent-child vegetable dish intake. Several cross-sectional studies in Western countries have demonstrated a similarity in the consumption of healthy foods and beverages between parents and their children when eating more meals together (19–22). In the present study, most study participants consumed meals together in the children’s cafeteria, suggesting a higher frequency of eating together. This suggests that parental dietary behaviors influence children’s eating habits through social, physical, and individual environmental factors (3). Additionally, children can play a role in changing parental dietary behaviors (23). This suggests that nutritional education for both children and parents may improve overall dietary habits, including vegetable intake.

Skin carotenoid levels were higher in the children than in their parents (means 366.8 in children and 315.0 in parents) (see Table 1). Additionally, the number of well-balanced meals was higher among children; the proportion of those who ate well-balanced meals twice or more times/day was 77.6 % in children and 41.0 % in parents (see Table 1). Takeuchi et al. reported that the median skin carotenoid level, measured using Veggie Meter^®^, was 335 (interquartile range: 227–407) in 10-year-old schoolchildren in Japan (*n* = 315) (24). Takayanagi et al. (25) reported that the mean skin carotenoid level, measured using Veggie Meter^®^, among men and women without metabolic syndrome (mean age: 57.4 years) in Japan was 377.3 (SD: 122.8; *n* = 1661) (25). The skin carotenoid levels in children in the present study were higher than those reported previously (24), while those in adults were lower (25). The nature of the study area (children’s cafeterias) may explain this difference. Children’s cafeterias provide meals to disadvantaged children, eliminate meals taken in isolation, offer education using nutritious foods, and create spaces for community interaction (13, 26); their users include both low- and middle-to-high-income households. In the present study, 18% of households reported not being financially well-off. Horikawa et al. reported that children from low-income households tended to have higher nutrient deficiency rates for nutrients that are found in high amounts in vegetables (vitamin B6, pantothenic acid, potassium, magnesium, phosphorus, iron, and zinc) than did those from middle-income households on days without school lunches (27). Well-balanced school lunches may significantly contribute to higher levels of skin carotenoids in children from low-income households.

The positive correlation between parent-child skin carotenoid levels was significantly attenuated after adjusting for smoking status and BMI, which are known to reduce skin carotenoid levels (28). Conversely, the positive correlation between parent-child intake of vegetable dishes persisted even after accounting for these factors. This suggests that parent-child dietary behaviors may be similar; however, unhealthy lifestyles, such as smoking and obesity, could counteract the benefits of increased vegetable intake through metabolic processes within the body.

This is a limited report examining the parent-child resemblance in skin carotenoid levels measured using RS. Surveys were conducted separately for parents and children. However, this study had several limitations. This was a cross-sectional study, meaning causal inferences cannot be drawn. Additionally, we were unable to assess the influence of dietary changes among parents on their children’s dietary behaviors. Detailed dietary surveys were also not conducted. We measured the number of vegetable dishes consumed using a self-administered questionnaire. The number of vegetable dishes consumed was determined by assessing how many dishes of 70 g servings were consumed (17, 18); although this method is employed for self-assessment of dietary intake based on the Japanese Food Guide Spinning Top, it is only a rough evaluation of intake amounts. In the present study, the association between skin carotenoid levels and vegetable dish intake was not clear for either children or parents. This suggests that fruit intake, which is also related to skin carotenoid levels, should have been assessed. Unfortunately, we did not measure fruit intake. Additionally, we did not evaluate the type of dietary supplement use. Skin carotenoid levels are also affected by internal factors such as smoking status and BMI (28). In the present study, the positive correlations between parental and child skin carotenoid levels were attenuated after adjusting for parental smoking status and BMI, but not for children’s body weight and height, which should have been accounted for. The information on parental weight and height was self-reported, which may introduce reporting bias. Additionally, we excluded participants without skin carotenoid data; many children came to the children’s cafeteria alone, and we could not measure skin carotenoid levels for their parents, meaning we cannot exclude the possibility of selection bias. Furthermore, the sample size was determined without a priori power analysis. A post-hoc power analysis revealed a power of 94.1 %, indicating that the study was adequately powered to detect significant effects. Lastly, as this study was performed on a Japanese population, the generalizability of the findings to other populations is unknown; further studies are needed to assess parent-child relationships in skin carotenoid levels in larger populations.

In conclusion, this is the first study to examine a correlation between parental and child skin carotenoid levels after adjusting for potential confounding factors. We found a positive correlation between parental and child skin carotenoid levels; however, the positive correlation was attenuated after adjusting for smoking status and BMI. We identified a positive correlation between parent-child vegetable dish intakes. This suggests that parent-child dietary behaviors may be similar; however, internal factors, such as smoking and obesity, could affect the skin carotenoid levels through metabolic processes within the body. Depending on the objective, it is necessary to carefully consider selection of the method for investigating vegetable intake such as skin carotenoid levels or questionnaires. Future studies should assess not only vegetable intake but also fruit intake, and they should also evaluate the types of supplements used by those who take supplements.

## Data Availability

The raw data supporting the conclusions of this article will be made available by the authors, without undue reservation.
